# S13-2 VARCITIES puts the citizens and the 'human community' in the eye of the future cities’ vision

**DOI:** 10.1093/eurpub/ckad133.064

**Published:** 2023-09-11

**Authors:** Denia Kolokotsa, Elisavet Tsekeri, Katerina Lilli

**Affiliations:** Technical University of Crete; Technical University of Crete; Technical University of Crete

## Abstract

In an increasingly urbanized world, local governments and international institutions strive to increase the productivity and efficiency of cities, recognized as economic growth hubs, as well as ensure a better quality of life and better living conditions for citizens. To meet these challenges, VARCITIES puts the citizen and the “human community” in the eye of the future cities’ vision. The aim of the VARCITIES project is to translate visionary ideas into real solutions by reshaping shared public spaces and making cities livable and welcoming.

Seven municipalities that are exposed to diverse climatic conditions and challenges around Europe have been identified as case studies. Each pilot city has recognized a large-scale site for co-designing and implementing local actions and has adopted an innovative cross-sectoral approach by combining urban digital transformation with nature-based actions. This innovative solution's implementation is consistent with the increase in citizens' awareness, respect for public spaces, and the integration of green spaces into everyday life. Developing a healthy green mindset for citizens and improving economic opportunities through green-digital strategies, drives the implementation of additional actions towards becoming a more sustainable and equitable city. The development process of these visionary ideas into feasible actions is approached by following a bottom-up planning and co-design participatory process involving local stakeholders, assuming a “multiple benefits” perspective, and also addressing social issues and cultural diversion.

